# Primary Aldosteronism Masked by Accessory Renal Arteries: A Case Report

**DOI:** 10.3390/jcm11216276

**Published:** 2022-10-25

**Authors:** Changqiang Yang, Xiangyu Yang, Si Wang, Xiaoping Chen, Kai Liu

**Affiliations:** Department of Cardiology, West China Hospital, Sichuan University, 37 Guoxue Road, Chengdu 610041, China

**Keywords:** primary aldosteronism, aldosterone-to-renin ratio, accessory renal arteries, secondary endocrine hypertension, aldosterone-producing adenoma

## Abstract

Primary aldosteronism (PA) is the most frequent form of secondary endocrine hypertension, which is characterized by excessive aldosterone secretion and suppressed renin. The currently recommended diagnostic algorithm is very clear, and the plasma aldosterone-to-renin ratio (ARR) is considered the first-line screening test. However, this indicator is influenced by many factors, some of which may cause false-negative results, consequently leading to underdiagnosed PA. Here, we report the rare case of a 38-year-old man who presented with bilateral accessory renal arteries and aldosterone-producing adenoma but had a negative ARR test result.

## 1. Introduction

Substantial evidence shows that primary aldosteronism (PA) is the most frequent cause of secondary endocrine hypertension and is closely related to cardiovascular risk [1,2]. The prevalence of PA in patients with newly diagnosed hypertension is at least 4% [3]. Moreover, the overall prevalence of PA increases with the severity of hypertension, from 3.9% in stage 1 hypertension to 11.8% in stage 3 hypertension [4]. Patients with PA more frequently present with hypertension-mediated organ damage and cardiovascular events when compared to age- and sex-matched patients with essential hypertension and the same degree of blood pressure elevation [5,6,7]. The currently recommended diagnostic algorithm is very clear, and the plasma aldosterone-to-renin ratio (ARR) is considered the first-line screening test in the current guidelines [1,2]. However, the diagnostic algorithm can be challenging due to several factors that are known to affect the secretion of renin and aldosterone, such as medications, serum potassium, and so on [8,9,10]. In addition, the existence of other causes of secondary hypertension can also interfere with the interpretation of ARR and thus hamper the diagnosis of PA [11,12,13]. Here, we report the first case of a patient with bilateral accessory renal arteries and an aldosterone-producing adenoma. High renin secretion from the accessory renal arteries converted higher ARR into normal ARR, thus obscuring the diagnosis of PA. The aldosterone-producing adenoma was confirmed by lateralization on adrenal vein sampling (AVS) and cured by unilateral adrenalectomy.

## 2. Case Description

A 38-year-old man presented with a history of hypertension for 4 years, and his highest blood pressure was 180/120 mmHg. He did not mention any discomfort such as dizziness, palpitations, or chest pain, except for throbbing headaches. He was treated with sacubitril/valsartan and amlodipine, but the blood pressure was controlled insufficiently and ranged from 140–160/100–110 mmHg. He complained of exertional fatigue and nocturia for the last six months. He visited the department of cardiology due to poor blood pressure control. In order to establish the etiology of hypertension, he was asked to stop previous antihypertensive medications and was administered diltiazem and terazosin. After taking diltiazem with terazosin for 2 weeks, the patient was admitted to our hospital for further examination.

His vital signs on admission were as follows: heart rate (HR) 82 times/minute; blood pressure (BP), 173/117 mmHg; body mass index (BMI), 30.99 kg/m^2^. Physical examination showed no significant findings. There was no proteinuria on dipstick testing, and the urinary albumin-to-creatinine ratio was 45.5 mg/g (reference: <30 mg/g). The serum potassium concentration was 2.8 mmol/L, and creatinine was 96 μmol/L (estimated glomerular filtration rate [eGFR] 86.03 mL/min/1.73 m^2^). The 24 h urinary potassium was 76.45 mmol/24 h, and sodium was 242.0 mmol/24 h, suggesting abnormally increased urinary potassium. Additionally, the 24 h urinary aldosterone concentration was elevated at 56.60 μg/24 h (reference: 1.19–28.1 μg/24 h). The results of other routine laboratory tests were within the normal range. The results of endocrine examination, including the levels of serum adrenocorticotropic hormone (ACTH), cortisol circadian rhythm, catecholamines, thyroid-stimulating hormone (TSH), free triiodothyronine (FT3), free thyroxin (FT4), and 24 h urinary metanephrine and normetanephrine, were all within the normal range. 

**Figure 1:**
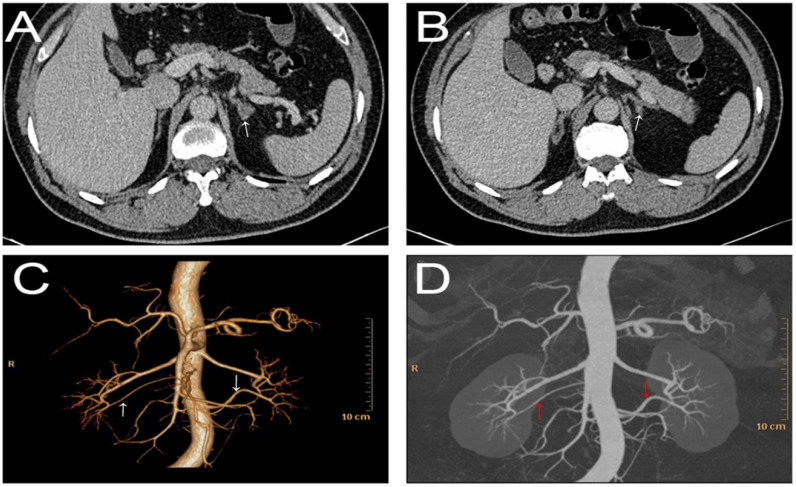
Adrenal contrast-enhanced CT. (**A**) The white arrow showed a round soft-tissue density mass with a diameter of 8 mm; (**B**) The white arrow showed the left inner branch adrenal gland was thickened. CTA showed no stenosis of the bilateral renal arteries and the presence of bilateral renal accessory arteries (pointed by the white arrow in (**C**) and red arrow in (**D**)).

On imaging examination, ultrasound echocardiography indicated cardiac hypertrophy (interventricular septum, 15 mm; left ventricular (LV) end-diastolic diameter, 49 mm; LV posterior wall thickness, 15 mm; LV mass index, 145 g/m^2^; LV ejection fraction, 61%). Carotid ultrasonography demonstrated left carotid intima-media thickening. These findings suggested hypertension-mediated organ damage. Ultrasonography of the renal artery showed no abnormality, and carotid-femoral pulse wave velocity was also within the normal range. Fundoscopic examination showed no hypertensive or atherosclerotic changes in the retina. Technetium-99m diethylene triamine pentaacetic acid (Tc-99m DTPA) renal dynamic scintigraphy demonstrated symmetrical uptake bilaterally (left renal GFR = 39.1 mL/min and right renal GFR = 40.3 mL/min). Adrenal contrast-enhanced computed tomography (CT) showed that the left inner branch adrenal gland was thickened and had a round soft-tissue density mass with a diameter of 8 mm (Figure 1). In addition, renal CT angiography showed no stenosis of the renal arteries, but rather the presence of bilateral accessory renal arteries (Figure 1).

Based on these findings, we suspected that the hypertension might be caused by the mass in the left adrenal gland. Therefore, we performed two consecutive ARR measurements, as shown in Table 1. The patient was in a supine position overnight, and blood samples were collected at 8:00 in the supine state and the midmorning, after the patient had been up (sitting, standing, or walking) for at least 2 h. In the first ARR screening, the result was normal, and the synchronous serum potassium was 2.95 mmol/L. To eliminate the influence of hypokalemia on ARR measurement, he was administered 10% potassium chloride solution (90–120 mL/day) orally for 4 days. When the serum potassium rose to 3.53 mmol/L, we conducted the second screening test. The result still showed that the ARR was in normal range.

The clinical characteristics were summarized as follows: poor blood pressure control, hypertension concomitant with refractory hypokalemia, and left adrenal gland mass in the CT scan. Although the patient showed consistently non-suppressed renin and negative ARR, we could not completely exclude the possibility that the adrenal gland mass was not an aldosterone-producing adenoma (APA), and the clinical suspicion of PA remained high. Therefore, we performed selective renal venous sampling and adrenal venous sampling (AVS). The patient was placed in a supine position and was on a normal diet. Catheters for venous sampling were placed through a femoral vein puncture. Blood samples were collected from the right renal vein, left renal vein, and the distal end of the inferior vena cava. Blood samples from the left and right renal veins showed elevated renin concentration, as shown in Table 2. AVS with ACTH stimulation was performed. A continuous cosyntropin infusion (50 µg/h started 30 min before sampling) was used for stimulation. Blood samples were collected from the right adrenal vein (AV), left AV, and distal end of the inferior vena cava. The results of AVS were shown in Table 3. The ratio of aldosterone/cortisol in the left AV to aldosterone/cortisol in the right AV was 4.78, which was >4, suggesting excessive secretion of aldosterone from the left adrenal gland according to the consensus on the use of AVS for the subtyping of primary aldosteronism [14].

**Figure 2:**
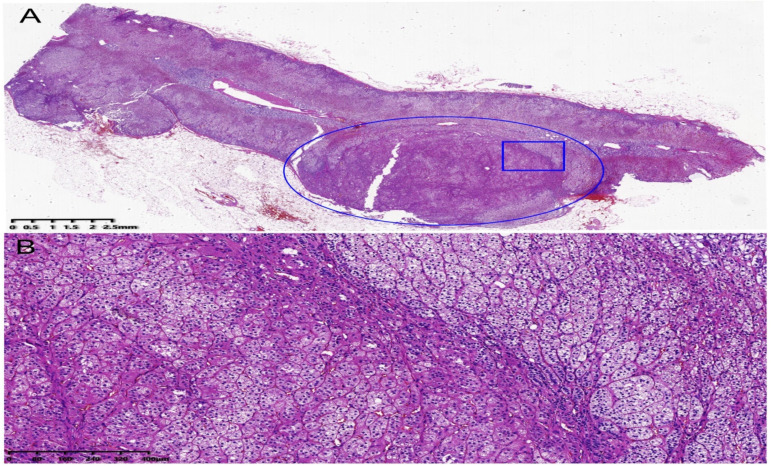
Histopathologic pictures of left adrenal gland. (**A**) Hematoxylin–eosin staining for left adrenal gland; the blue oval showed the left adrenal adenoma; the blue rectangle part of gland in (**A**) was magnified as shown in (**B**). (**B**) Hematoxylin–eosin staining showed fasciculata (ZF)-like cells, which were large, lipid laden clear, with round to oval vesicular nuclei, and zona glomerulosa (ZG)-like cells, characterized as small, compact cells, with high nuclear/cytoplasmic ratio and moderate amount of lipid.

We strongly suspected that the left adrenal gland mass was an APA. After the patient’s informed consent was obtained, laparoscopic left adrenalectomy was performed. Histopathologic findings were suggestive of adrenal adenoma (Figure 2A,B). After surgery, he was administered diltiazem and terazosin. In the 1-month follow up, the serum potassium concentration was 4.81 mmol/L without supplementation of potassium chloride, and the blood pressure was uncontrolled at 160–180/100–110 mmHg. At the same time, we performed the upright aldosterone–renin ratio test. Although the serum aldosterone concentration returned to normal, the peripheral renin concentration was more markedly elevated than before, as shown in Table 1. In addition, the 24 h urinary aldosterone concentration also returned to normal at 8.74 μg/24 h (reference: 1.19–28.1 μg/24 h). Therefore, we replaced diltiazem and terazosin with olmesartan/amlodipine. After 2 months, his blood pressure was controlled at 120–130/79–80 mmHg with olmesartan (20 mg)/amlodipine (5 mg)/day.

## 3. Discussion

In this report, we have described a patient with hypertension with concomitant refractory hypokalemia and excessive aldosterone secretion but negative ARR test results, even after following the recommended pathways of the screening test to prevent false-negative results. In addition, computed tomography angiography (CTA) did not show stenosis of the renal arteries, but rather demonstrated the presence of bilateral accessory renal arteries and a left adrenal gland mass. Due to high clinical suspicion of PA, further investigations were performed. The left adrenal gland mass was confirmed as an APA by lateralization on AVS and treated by unilateral adrenalectomy. At the same time, bilateral renal vein sampling revealed elevated renin concentration before surgery, while the peripheral renin concentration was more elevated after surgery than before it. Therefore, we concluded that the bilateral accessory renal arteries were the cause of hyperreninemia, which masked the typical characteristics of PA, leading to a negative ARR screening result in this patient.

In this patient, it is important to exclude some factors that typically contribute to a false-negative ARR. First, a common cause is dietary salt restriction [2]. However, this patient had a liberalized sodium intake, and his 24 h urinary sodium excretion was 242 mmol/day without evidence of dietary sodium restriction. Therefore, we could rule out the possibility that low dietary sodium intake led to the false-negative ARR result in this patient. Second, some medications can also interfere with the measurement of ARR and lead to false negative results [2]. In this patient, the washout time of all interfering antihypertensive medications (sacubitril/valsartan and amlodipine) was beyond 2 weeks according to the clinical practice guidelines for the management of patients with primary aldosteronism [2]. Therefore, we could exclude the possibility of these antihypertensive medications interfering with the measurement of ARR. Another well-known cause of false-negative ARR is renal artery stenosis (RAS) [12]. In this patient, RAS was carefully excluded by renal CTA and Tc-99m DTPA renal dynamic scintigraphy. In addition, high plasma renin activity may also occur with malignant hypertension [15]. However, patients with malignant hypertension often present with progressive hypertension-mediated organ damage, such as advanced retinopathy and renal damage, neither of which were observed in this patient. Finally, the possibility of reninoma should be considered despite the rarity of this disease [16]. Renal CT with contrast injection has almost 100% sensitivity for the detection of reninoma [17,18], and renal vein sampling also has 50% to 60% sensitivity for detecting renin lateralization [18]. In this patient, the adrenal contrast-enhanced CT showed no tumors in the renal parenchyma and renal vein sampling also showed an absence of lateralization of direct renin secretion. Therefore, we could completely exclude a renin-secreting tumor of the juxtaglomerular apparatus.

After excluding these interfering factors and concomitant diseases, it seemed that the patient indeed presented with an aldosterone-producing adenoma with non-suppressed renin. Jansen et al. [19]. reported a case series of seven patients with non-suppressed renin levels after excluding the common factors leading to false-negative ARR results. However, could there be a pathophysiological mechanism to explain this phenomenon? To date, there is no evidence in the published literature that the non-suppressed renin in PA is attributable to physiological factors. However, the present patient was different from these patients and had bilateral accessory renal arteries on CT angiography.

Are bilateral accessory renal arteries related to the non-suppressed renin or renin-dependent hypertension? A retrospective analysis of 3000 patients’ renal artery CT imaging data showed that the accessory renal artery (ARA) was a risk factor for hypertension [20]. In fact, as early as 2005, David et al. proposed a new syndrome of renin-dependent hypertension caused by non-stenotic accessory renal arteries [21]. One hypothesis put forward to explain the mechanism was Hagen–Poiseuille’s Law (ΔP = 8 μLQ/πR4), where P = pressure, μ = viscosity, L = length of vessel, and Q = volume flow, which described the pressure drop along a cylindrical tube as being directly proportional to its length and inversely proportional to the tube’s radius to the fourth power [21]. It was, therefore, possible that the longer and narrower the caliber of the accessory renal artery, the greater the drop in the blood pressure as blood flows from the proximal to the distal end, leading to under perfusion of the affected renal segment and activation of the renin–angiotensin system, which results in renin-dependent systemic hypertension [22]. 

Although no studies have evaluated to what degree a difference in the pressure gradient between the aorta and distal segment of an ARA might influence the production of renin, studies on RAS have demonstrated that a Pd/Pa ratio (the ratio of distal renal pressure to aortic pressure) of 0.90 could be considered the threshold value below which the stenosis was likely responsible for a significant upregulation of renin production [23]. In addition, a previous study showed that the peripheral plasma renin activity was higher in the group with an accessory renal artery compared to a matched control group of hypertensive patients without an accessory renal artery [24]. Recently, a study found that the plasma renin concentrations were significantly higher in hypertensive patients with an accessory renal artery compared to those without [25]. Taken together, an accessory renal artery was potentially associated with high renin concentration and might be an overlooked cause of renin-dependent hypertension. 

In this patient, renal CTA demonstrated two elongated, narrow-caliber accessory renal arteries arising from the abdominal aorta that fed the lower pole of the right and left kidneys. Thus, we speculated that hypo-perfusion of the affected renal segment could lead to segmental hyperreninemia according to the theory of Hagen-Poiseuille’s Law. In addition, blood samples from both the left and right renal veins confirmed the bilaterally elevated renin concentrations. After laparoscopic resection of the left adrenal gland, the peripheral blood renin concentrations were more markedly elevated than before. Therefore, we demonstrated that the non-suppressed renin in this PA patient was attributable to high renin secretion from the bilateral accessory renal arteries. 

Clinicians should cautiously interpret the aldosterone–renin ratio if hyperaldosteronism is highly suspected, especially in the background of other causes of secondary hypertension. The utility of AVS as an initial diagnostic test is “never” mentioned in the current guidelines [2]. The ancillary diagnostic test of intra-procedural renal vein sampling is now seldom recommended by the current guidelines. However, in patients with concomitant accessory renal arteries with high renin status, the above-mentioned diagnostic tests are very important to distinguish between right and left adrenal venous aldosterone and renal venous renin concentrations. Therefore, it was appropriate to conduct AVS and renal venous sampling in this patient despite the low ARR, which could identify masked PA due to high renin secretion from the bilateral accessory renal arteries. There are some limitations of our study. As the phenomenon of secondary hyperaldosteronism was indicated by postural stimulation tests, we did not perform confirmatory testing (saline suppression and captopril test). Additionally, immunochemistry and genetic testing related to aldosterone-producing adenoma was not performed. There was also a lack of available interventions to correct the postulated hypoperfusion caused by the accessory renal arteries.

## 4. Conclusions

An accessory renal artery may be an overlooked cause of renin-dependent hypertension, which can obscure the underlying disorder of PA with concurrent accessory renal artery. A negative ARR screening result alone cannot exclude PA. If clinical suspicion remains high, a comprehensive evaluation should be undertaken to avoid a missed diagnosis. AVS and renal venous sampling can be performed to differentiate masked PA due to high renin secretion from other causes such as reninoma, renal artery stenosis, and accessory renal artery.

## Figures and Tables

**Table 1:** Postural stimulation tests.

Variable	Serum Potassium (mmol/L) (Reference: 3.5–5.3)	Renin (μIU/mL)	Aldosterone (ng/dL)	ARR {(ng/dL)/(μIU/mL)} (Reference: <3.7)
First screening test	2.95			
Supine position		26.61 (reference: 2.8–39.9)	22.8 (reference: 3–23.6)	0.86
Upright position		118.2 (reference: 4.4–46.1)	39.7 (reference: 3–35.3)	0.34
Second screening test	3.53			
Supine position		50.56 (reference: 2.8–39.9)	47.6 (reference: 3–23.6)	0.94
Upright position		50.22 (reference: 4.4–46.1)	45.9 (reference: 3–35.3)	0.91
1 month after surgery	4.81			
Upright position		121.4 (reference: 4.4–46.1)	11.9 (reference: 3–35.3)	0.1

**Table 2:** Bilateral renal vein sampling.

Variable	Inferior Vena Cava	Right Renal Vein	Left Renal Vein
Renin (μIU/mL)	41.80	72.54	66.95
Aldosterone (ng/dL)	53.10	44.10	43

**Table 3:** Selective adrenal vein sampling.

Variable	Aldosterone (ng/dL)	Cortisol (nmol/L)	Selectivity Index (SI)	Aldosterone/ Cortisol Ratio	Lateralization Index
ACTH Stimulation					
Inferior Vena Cava	47.20	654			
Right Adrenal Vein	1050	32856	50.2	0.0319	4.78
Left Adrenal Vein	4960	32411	49.5	0.1530	

## Data Availability

All authors confirm that all the data that support the findings of this report are available on request from the corresponding author.
